# Effect of Deferasirox on Shunt Fraction During Thoracic Surgery With One-Lung Ventilation: A Randomized Controlled Study

**DOI:** 10.7759/cureus.39071

**Published:** 2023-05-16

**Authors:** Rajesh Raman, Parvathy S Nair, Ahsan Khaliq Siddiqui, Rati Prabha, Monica Kohli, Vinod K Srivastava

**Affiliations:** 1 Department of Anaesthesiology, King George's Medical University, Lucknow, IND

**Keywords:** hypoxic pulmonary vasoconstriction, general thoracic surgery, shunt fraction, one-lung ventilation, deferasirox

## Abstract

Context

Deferasirox, an iron chelator, can potentially reduce intraoperative right-to-left shunt and improve oxygenation in patients undergoing thoracic surgery requiring one-lung ventilation (OLV) by potentiating hypoxic pulmonary vasoconstriction (HPV).

Aim

The aim was to determine the effect of deferasirox on the intraoperative shunt fraction (SF) of patients undergoing thoracic surgery using OLV.

Study design and settings

This was a prospective, single-blind, randomized, controlled study. The study was conducted at a tertiary-care hospital.

Methods

Before surgery, 64 patients were allocated to two groups comprising 32 patients each. Group D patients were administered deferasirox, while those in group C were given a placebo. We included patients with the American Society of Anesthesiologists physical status III or IV, aged 18-60 years, undergoing elective thoracic surgery needing OLV. SF was the primary outcome variable. Secondary outcome variables were arterial oxygen tension (PaO_2_), peripheral oxygen saturation (SpO_2_), the ratio of PaO_2_ and inspired oxygen concentration (P/F), and complications such as desaturation episodes, hypotension, and tachycardia.

Results

Baseline and postoperative values of outcome variables were statistically similar in both groups. Intraoperative values of SF were lower and PaO_2_, SpO_2_, and P/F were higher in group D. The incidence of intraoperative desaturation was lower in group D.

Conclusion

We conclude that pre-treatment with deferasirox reduces intraoperative SF and improves oxygenation during thoracic surgery using OLV.

## Introduction

The frequency of hypoxemia in thoracic surgeries that necessitate one-lung ventilation (OLV) varies between 5% and 10% [1]. Extracardiac right-to-left shunt (RLS) resulting from perfusion of the non-dependent lung is the predominant mechanism responsible for intraoperative hypoxemia during OLV [2,3]. Hypoxic pulmonary vasoconstriction (HPV) is the primary physiologic mechanism responsible for reducing the blood flow to the non-ventilated lung and reducing RLS. Without HPV, the shunt would range from 40% to 60% due to unrestricted blood flow to the non-ventilated lung and cause hypoxemia in all the patients with OLV. HPV decreases perfusion of the non-ventilated lung by approximately 50%, reducing RLS and the incidence of hypoxemia [4]. Administration of iron before hypoxemia has been shown to reduce HPV [5,6]. At the same time, HPV is potentiated by deferoxamine, an iron chelator [6]. Therefore, deferasirox, an orally active, highly selective iron chelator, can potentially enhance HPV and reduce RLS during OLV. This study aimed to investigate how deferasirox affects shunt fraction (SF) in thoracic surgery with OLV. It was hypothesized that deferasirox reduces the intraoperative SF during thoracic surgeries requiring OLV.

## Materials and methods

This single-blind, randomized, parallel-arm, control trial was undertaken after it was approved by the ethics committee (Institutional Ethics Committee, King George's Medical University; reference number: V-PGTSC-IIA/P45) and registered with the Clinical Trials Registry of India (registration number: CTRI/2022/04/041753). Informed and written consent was obtained from all the patients recruited in our trial. Patients of either gender, between 18 and 60 years, and having American Society of Anesthesiologists physical status III or IV planned for elective thoracic surgery with OLV were included in the trial. Patients with bilateral pulmonary disease, cardiovascular disease, renal or hepatic disease, obesity, and pregnancy were excluded from the trial.

The recruited participants were allocated one of the following groups using a sequentially numbered opaque sealed envelope technique: group D patients received 500 mg deferasirox tablet (Desirox 500, Cipla, Solan, India) dissolved in 100 milliliters of water; group C: patients received glucose tablets dissolved in 100 milliliters of water.

On the evening before surgery, the pre-anesthetic check-up of all the patients was reviewed. The anesthesiologist opened the sealed envelope in a separate room, dissolved deferasirox or glucose tablet, and gave it to the patient to drink. The participants were unaware of the study medication administered on the evening before surgery.

On patients' arrival in the operation theater, a pulse oximeter and electrocardiogram were applied. A 20-gauge cannula was placed in the radial artery to enable the collection of arterial blood gases (ABG) and to take invasive blood pressure measurements. One ABG sample was taken to measure arterial oxygen pressure (PaO_2_) and the ratio of PaO_2_ and the fraction of inspired oxygen (P/F) before anesthesia induction. After administering intravenous fentanyl (2 µg/kg), anesthesia was induced with intravenous propofol injection (1.5-2 mg/kg). In addition, an intravenous injection of 0.1 mg/kg vecuronium bromide was administered to aid the placement of a double-lumen endobronchial tube (Broncho-Cath, Mallinckrodt, Dublin, Ireland). The correct placement of the double-lumen tube was verified using a 4-mm diameter, 65-cm long, Flexible Intubation Video Endoscope set (Karl Storz SE & Company, Tuttlingen, Germany). Patients were ventilated with a tidal volume of 6-8 ml/kg, positive end-expiratory pressure (PEEP) of 5 mmHg, inspiratory to expiratory time ratio of 1:2, and respiratory rate of 12-14 per minute. End-tidal carbon dioxide (EtCO_2_) was maintained between 35 and 45 mmHg. For OLV, the settings were as follows: tidal volume: 5-6 ml/kg, PEEP: 5 mmHg, and respiratory rate: 12-14 per minute with a target EtCO_2_ of 35-45 mm Hg. The tidal volume and respiratory rate were reduced if the peak airway pressure exceeded 35 cm H_2_O or plateau pressure exceeded 25 cm H_2_O. Anesthesia was maintained using oxygen, sevoflurane, and intermittent boluses of intravenous vecuronium. Patients were administered 100% oxygen throughout the surgery. After the induction of anesthesia, a central venous catheter (CVP) was placed in the right internal jugular vein. Intraoperative episodes of hypoxemia were managed by checking the correct position of the double-lumen tube, applying a recruitment maneuver to the ventilated lung, and intermittent reinflation of the non-dependent lung. Patients were extubated at the end of surgery after discontinuing anesthetic drugs and reversal of vecuronium.

SF was the primary outcome variable in the current study. Secondary outcome variables were PaO_2_, P/F, SpO_2_, and complications. SF was calculated at 10, 20, and 30 minutes of OLV and six hours after extubation. PaO_2_, P/F, and SpO_2_ were compared before induction of anesthesia (baseline), at 10, 20, and 30 minutes after the patient was on OLV, and six hours after extubation. Complications, including desaturation episodes, were measured until 24 hours after the start of surgery.

SF was obtained using the following equation [7]:

$SF= \left ( CcapO_{2}-CartO_{2} \right ) \div \left ( CcapO_{2}-CvenO_{2} \right )$.

Where CcapO_2_, CartO_2_, and CvenO_2_ are the oxygen content of pulmonary capillary, systemic artery, and mixed venous blood, respectively. These were obtained using the following equations:

$CartO_{2}= \left ( 1.36\times SaO_{2}\times Hb \right ) + \left ( 0.0031 \times PaO_{2} \right )$.

$CcapO_{2}= \left ( 1.36\times Hb \right ) + 0.0031 \times \left \{ FiO_{2}\left ( P_{B}-PH_{2}O \right ) -PaCO_{2}\div RQ \right \}$.

$CvenO_{2}= \left ( 1.36\times SvO_{2} \times Hb\right ) + \left ( 0.0031\times PvO_{2} \right )$.

Where P_B_ is atmospheric pressure (760 mmHg); PH_2_O is saturated vapor pressure at 37°C (47 mmHg); RQ is respiratory quotient (0.8); SaO_2_ and SvO_2_ are the arterial and central venous oxygen saturation, respectively; Hb is the hemoglobin of the patient; and PvO_2_ is oxygen tension in central venous blood. For CvenO_2_, blood from CVP was taken in place of the pulmonary artery.

Our study had a predetermined power of 0.8 and an alpha error of 0.05. SF had a standard deviation (SD) of 13% in a previous study [8]. With an SD of 13%, at least 27 patients were required in each study arm to detect a clinically significant difference of 10% in SF. We included 32 patients in each group to account for patient exclusion and data loss. Data were analyzed using SPSS version 26 (IBM Corp., Armonk, NY) for Windows. Categorical data were analyzed using Fisher's exact test and are presented as numbers (percentages). Continuous data were compared using unpaired t-test and are presented as mean ± SD. A two-sided p < 0.05 was considered statistically significant for all the statistical tests.

## Results

**Figure 1:**
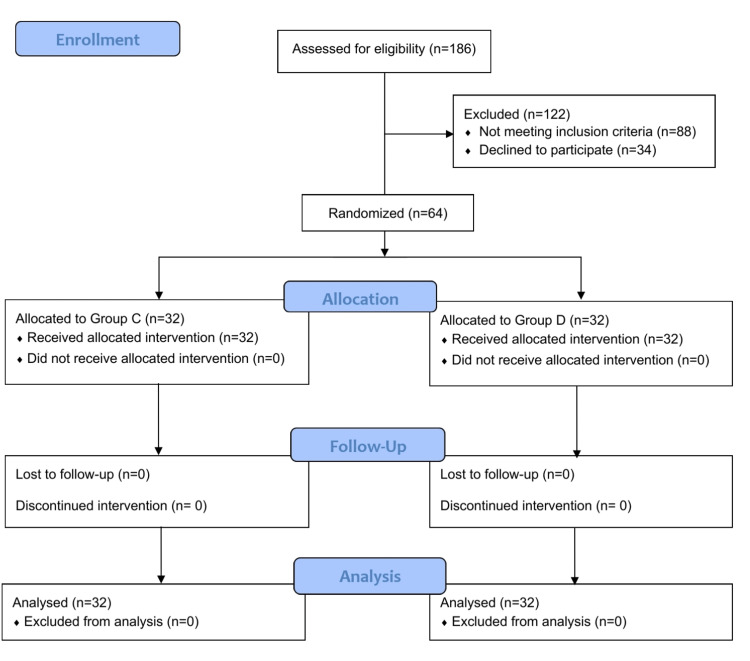
CONSORT diagram showing the flow of the patients in the trial CONSORT: Consolidated Standards of Reporting Trials.

Figure 1 shows the Consolidated Standards of Reporting Trials (CONSORT) diagram depicting the flow of patients in our trial. As shown in Table 1, baseline and demographic variables were statistically comparable between the groups. A comparison of SF, SpO_2_, PaO_2_, and P/F is shown in Table 2. SF was statistically lower in group D intraoperatively compared to group C at 10, 20, and 30 minutes. PaO_2_ and P/F were significantly higher in group D at 10, 20, and 30 minutes. SpO_2_ was comparable between the groups at all the points of observation. Complications are compared in Table 3. Desaturation episodes were significantly less frequent in group D. Post-hoc power analysis using SF yielded a power of >99% for all the time points SF was observed and compared in our study.

**Table 1:** Comparison of demographic and baseline characteristics of the two groups Data are presented as mean ± standard deviation or numbers (percentages). BMI: body mass index; ASA: American Society of Anesthesiologists physical status; kg: kilogram; m: meter; FVC: forced vital capacity; FEV_1_: forced expiratory volume in the first second; D_LCO_: diffusing capacity for carbon monoxide; VATS: video-assisted thoracic surgery.

Characteristics	Group C (n = 32)	Group D (n = 32)	p
Age (years)	34.50 ± 12.37	33.97 ± 10.02	0.851
Gender (male/female)	26 (81.25%)/6 (18.75%)	22 (68.75%)/10 (31.25%)	0.248
Height (meter)	1.60 ± 0.07	1.62 ± 0.06	0.936
Weight (kg)	64.34 ± 3.24	64.44 ± 2.77	0.901
BMI (kg/m^2^)	24.58 ± 2.23	24.54 ± 1.77	0.935
ASA III/IV	5 (15.63%)/27 (84.38%)	4 (12.50%)/28 (87.50%)	0.500
FVC	76.94 ± 6.66	75.94 ± 7.52	0.575
FEV_1_	64.00 ± 6.66	64.50 ± 6.97	0.770
FEV_1_/FVC	85.56 ± 9.40	85.89 ± 13.76	0.433
D_LCO_	67.19 ± 7.64	67.94 ± 8.61	0.714
Type of surgery			
Lobectomy	12 (37.50%)	10 (31.25%)	0.964
VATS	7 (21.88%)	8 (25.00%)	
Segmental resection	8 (25.00%)	9 (28.13%)	
Pneumonectomy	3 (9.38%)	2 (6.25%)	
Other surgeries	2 (6.25%)	3 (9.38%)	

**Table 2:** Comparison of shunt fraction and oxygenation at various time points in the study Data are presented as mean ± standard deviation, * statistically significant. PaO_2_: partial pressure of oxygen in arterial blood; P/F: ratio of PaO_2_ and the fraction of inspired oxygen; SpO_2_: oxygen saturation; mmHg: millimeters of mercury.

Outcome	Shunt fraction (%)			PaO_2 _(mmHg)		SpO_2 _(%)		P/F	
Group	C (n = 32)	D (n = 32)		C (n = 32)	D (n = 32)	C (n = 32)	D (n = 32)	C (n = 32)	D (n = 32)
Baseline	Not measured			66.16 ± 2.99	64.72 ± 3.60	96.69 ± 2.74	96.31 ± 2.72	66.16 ± 2.99	64.72 ± 3.60
				p = 0.088		p = 0.585		p = 0.088	
10 minutes	9.17 ± 0.67	5.88 ± 0.77		114.06 ± 15.62	125.12 ± 17.93	97.78 ± 1.31	98.00 ± 1.57	114.06 ± 15.62	125.12 ± 17.93
	p < 0.001*			p = 0.011*		p = 0.547		p = 0.011*	
20 minutes	24.94 ± 0.60	20.67 ± 0.45		122.69 ± 17.43	147.88 ± 32.70	98.16 ± 1.25	98.03 ± 1.60	122.69 ± 17.43	147.88 ± 32.70
	p < 0.001*			p < 0.001*		p = 0.728		p < 0.001*	
30 minutes	26.66 ± 0.69	21.37 ± 0.34		126.28 ± 10.68	161.19 ± 21.63	97.09 ± 2.07	96.72 ± 1.44	126.28 ± 10.68	161.19 ± 21.63
	p < 0.001*			p < 0.001*		p = 0.404		p < 0.001*	
6 hours	9.43 ± 0.41		9.55 ± 0.46	85.78 ± 16.56	93.72 ± 23.35	97.91 ± 1.39	93.30 ± 1.94	85.78 ± 16.56	93.72 ± 23.35
	p = 0.272			p = 0.122		p = 0.712		p = 0.122	

**Table 3:** Comparison of complications in the study Data are presented as numbers (percentages); * statistically significant.

Complications	Group C (n = 32)	Group D (n = 32)	p
Desaturation episodes	29 (90.63)	8 (25.00)	<0.001*
Hypotension	6 (18.75%)	8 (25.00%)	0.763
Tachycardia	9 (28.13%)	10 (31.25%)	1.000
Nausea/vomiting	9 (28.13%)	13 (40.63%)	0.430
Pneumothorax	1 (3.13%)	1 (3.13%)	1.000
Air leak	1 (3.13%)	1 (3.13%)	1.000

## Discussion

In the current trial, we studied the effect of deferasirox on SF of patients undergoing thoracic surgery with OLV. It was found that deferasirox reduced SF during OLV. Patients receiving deferasirox had better oxygenation with higher PaO_2_ and PF during OLV. SpO_2_ was similar between the groups during the perioperative period. Intraoperative episodes of desaturation were also fewer in patients receiving deferasirox.

RLS is inevitable when a patient is on OLV because the non-ventilated lung is perfused in the absence of ventilation [9]. RLS has a severe negative impact on the patient's oxygenation as the shunted blood does not get oxygenated. When mixed with the oxygenated blood of pulmonary veins, this deoxygenated blood results in systemic oxygen desaturation. This RLS is the predominant mechanism responsible for decreased saturation during OLV.

HPV is a reflex contraction of vascular smooth muscle in response to reduced regional oxygen tension. HPV diverts the blood from the less oxygenated regions to highly oxygenated regions of the lung, reducing the RLS and improving systemic oxygenation. HPV is predominantly governed by hypoxia-inducible factor (HIF). HIF, a hypoxia-mimetic agent, promotes HPV due to hypoxia and can even emulate hypoxia [5,10,11]. Degradation of HIF by proteosomes is an iron-dependent process and requires Fe (II) as an obligatory component [12,13]. Consequently, iron decreases HPV, and iron chelators are predicted to increase HPV and decrease SF.

The role of iron and its chelators for modulating hypoxia-induced pulmonary vascular vasoconstriction is well described in the scientific literature [14,15]. In a randomized control trial by Smith et al., the administration of iron hydroxide reversed the pulmonary hypertensive response mediated by HPV in response to hypoxia by 40%. In the same trial, iron deficiency achieved by multiple venesections caused a 25% increase in pulmonary artery systolic pressures by potentiating HPV [15]. In another crossover study by Smith et al., intravenous infusion of iron profoundly attenuated the acute rise of pulmonary artery systolic pressure in volunteers subjected to eight hours of hypoxia. When pre-treated with a single dose of deferoxamine, the same individuals exhibited an acute rise in pulmonary artery systolic pressure due to an increase in HPV [6]. The authors concluded that iron availability modulates the HPV response. In another study with a crossover design, acute infusion of a single dose of deferoxamine led to a significant rise in pulmonary vascular resistance. This observation was attributed to the stabilization of HIF and the potentiation of HPV by deferoxamine [5].

Although the potentiation of HIF and HPV can be harmful to patients with pulmonary hypertension, they can be advantageous for patients who are undergoing thoracic surgery with OLV. HPV reduces RLS and hence improves systemic saturation. We could not find any published literature studying the effect of iron or its chelators on patients undergoing thoracic surgery. Our findings of reduced SF and improved oxygenation (P/F and PaO_2_) imply that deferasirox, like deferoxamine, stabilizes HIF and potentiates HPV.

Despite improvement in intraoperative SF, and consequently in systemic PaO_2_, no improvement in SpO_2_ was observed in our trial. The relation between PaO_2_ and SpO_2_ is not linear [16]. The oxy-hemoglobin dissociation curve is sigmoid shaped, with the upper flat part having a minimal rise in SpO_2_ despite larger increases in PaO_2_. Furthermore, the SpO_2_ is limited to 100%, while PaO_2_ can be several times higher, as seen in our study. As most of the patients had SpO_2_ between 90% and 100%, despite a large difference in PaO_2_, SpO_2_ was statistically similar in our study.

Intraoperative desaturation episodes were much lower in patients pre-treated with deferasirox. Unfortunately, we do not have similar trials to compare this. However, this can be attributed to improved oxygenation in the patients given deferasirox due potentiation of HPV and reduced RLS.

There are three limitations of our trial. First, the single-center design of our trial limits the generalizability to other populations. Second, we used a blood sample from the central vein instead of a pulmonary artery blood sample. This was done to reduce the complications associated with pulmonary artery catheter placement. However, this may have introduced some inaccuracies with SF calculation in our trial. The third limitation was the small sample size of our study.

## Conclusions

Based on our trial studying the effect of deferasirox on SF, we conclude that deferasirox reduces left-to-right shunt, improves systemic oxygenation, and reduces desaturation episodes in patients undergoing thoracic surgery with OLV. Further research is needed to confirm the findings of this study and investigate the effects of deferasirox on a larger sample of patients.
